# CAR expression in invasive breast carcinoma and its effect on adenovirus transduction efficiency

**DOI:** 10.1186/s13058-024-01880-z

**Published:** 2024-09-10

**Authors:** Abraham T. Phung, Jaimin R. Shah, Tao Dong, Tony Reid, Christopher Larson, Ana B. Sanchez, Bryan Oronsky, William C. Trogler, Andrew C. Kummel, Omonigho Aisagbonhi, Sarah L. Blair

**Affiliations:** 1 Moores Cancer Center, University of California San Diego, La Jolla, CA 92037 USA; 2 Department of Chemistry and Biochemistry, University of California San Diego, La Jolla, CA 92037 USA; 3 Department of NanoEngineering, University of California San Diego, La Jolla, CA 92037 USA; 4 Materials Science and Engineering, University of California San Diego, La Jolla, CA 92037 USA; 5 EpicentRx, Inc, La Jolla, CA 92037 USA; 6 Department of Pathology, University of California San Diego, La Jolla, CA 92037 USA; 7 Department of Surgery, University of California San Diego, La Jolla, CA 92037 USA

## Abstract

**Background:**

Breast cancer is the second leading cause of death in women, with invasive ductal carcinoma (IDC) and invasive lobular carcinoma (ILC) as the two most common forms of invasive breast cancer. While estrogen receptor positive (ER+) IDC and ILC are treated similarly, the multifocality of ILC presents challenges in detection and treatment, worsening long-term clinical outcomes in patients. With increasing documentation of chemoresistance in ILC, additional treatment options are needed. Oncolytic adenoviral therapy may be a promising option, but cancer cells must express the coxsackievirus & adenovirus receptor (CAR) for adenoviral therapy to be effective. The present study aims to evaluate the extent to which CAR expression is observed in ILC in comparison to IDC, and how the levels of CAR expression correlate with adenovirus transduction efficiency. The effect of liposome encapsulation on transduction efficiency is also assessed.

**Methods:**

To characterize CAR expression in invasive breast carcinoma, 36 formalin-fixed paraffin-embedded (FFPE) human breast tumor samples were assayed by CAR immunohistochemistry (IHC). Localization of CAR in comparison to other junctional proteins was performed using a multiplex immunofluorescence panel consisting of CAR, p120-catenin, and E-cadherin. ILC and IDC primary tumors and cell lines were transduced with E1- and E3-deleted adenovirus type 5 inserted with a GFP transgene (Ad-GFP) and DOTAP liposome encapsulated Ad-GFP (DfAd-GFP) at various multiplicities of infection (MOIs). Transduction efficiency was measured using a fluorescence plate reader. CAR expression in the human primary breast carcinomas and cell lines was also evaluated by IHC.

**Results:**

We observed membranous CAR, p120-catenin and E-cadherin expression in IDC. In ILC, we observed cytoplasmic expression of CAR and p120-catenin, with absent E-cadherin. Adenovirus effectively transduced high-CAR IDC cell lines, at MOIs as low as 12.5. Ad-GFP showed similar transduction as DfAd-GFP in high-CAR IDC cell lines. Conversely, Ad-GFP transduction of ILC cell lines was observed only at MOIs of 50 and 100. Furthermore, Ad-GFP did not transduce CAR-negative IDC cell lines even at MOIs greater than 100. Liposome encapsulation (DfAd-GFP) improved transduction efficiency 4-fold in ILC and 17-fold in CAR-negative IDC cell lines.

**Conclusion:**

The present study demonstrates that oncolytic adenoviral therapy is less effective in ILC than IDC due to differences in spatial CAR expression. Liposome-enhanced delivery may be beneficial for patients with ILC and tumors with low or negative CAR expression to improve adenoviral therapeutic effectiveness.

**Supplementary Information:**

The online version contains supplementary material available at 10.1186/s13058-024-01880-z.

## Introduction

Invasive ductal carcinoma (IDC) and invasive lobular carcinoma (ILC) are the two most commonly occurring types of invasive breast cancer. IDC makes up 70–80% of breast cancer cases while ILC makes up only about 8–15% of breast cancer cases [1]. Compared to IDC, ILC is more difficult to detect by mammography due to its diffuse growth pattern, slow proliferation, and ill-defined margins [2, 3]. Therefore, ILC presents more frequently in older patients with larger tumors, later stage disease, and more nodal involvement [4–7], leading to poorer long-term outcomes [1, 4–8]. Patients with ILC are also at higher risk of late recurrences and bone metastasis, than those with similar grade and stage IDC [2, 9].

Oncolytic viral therapy is an emerging treatment option for a variety of cancers, including breast cancers [10]. Currently, adenoviruses remain one of the safest, most efficient and robust viral platforms to engineer gene therapy vectors and oncolytic viruses [11, 12]. In the last decade, several experimental oncolytic adenoviruses have been developed to treat breast [13, 14], gastrointestinal [15, 16], ovarian [17, 18], and brain cancers [19, 20]. Some oncolytic adenoviruses have completed Phase I clinical trials, including AdAPT-001, OBP-301, VCN-01, and ICOVIR-5 [21–24]. Oncolytic adenoviral therapy relies on receptor-based transduction through the coxsackievirus & adenovirus receptor (CAR) for most serotypes to infect, replicate inside of, and lyse cancer cells [11, 12, 25, 26]. Therefore, CAR expression in tumors affects adenoviral transduction efficiency.

Liposome encapsulation of oncolytic adenoviruses enables the virus to enter and transduce cancer cells without CAR expression on the surface of their membranes [27–30]. When the adenovirus is encapsulated in a liposome, the virus can enter the cell through membrane fusion, thereby bypassing the need for CAR-mediated entry [28–30]. Other serotype adenoviruses, such as Ad3, and chimeric adenoviruses, such as Ad5F35, can also bypass CAR-mediated entry; however, their transduction efficiency is still limited by the expression of other receptors, such as CD46 in the case of Ad3 and Ad5F35, and require additional fiber protein modifications to overcome these limitations [31]. The advantage of liposome-enhanced delivery of adenoviruses is that the transduction efficiency is independent of fiber receptor expression [32, 33]. Studies in pancreatic, liver, ovarian, and lung cancer models have demonstrated that liposome encapsulation improves the transduction of adenovirus [34–37].

IDC typically demonstrates moderate to high levels of CAR, and there are some pre-clinical data on the effects of adenoviral therapy on IDC models. However, there are limited studies on CAR expression and the effect of adenoviral therapy in ILC models [38–41]. The current study aimed to evaluate the extent to which CAR expression is observed in ILC in comparison to IDC and to assess how the levels of CAR expression correlate with adenovirus transduction efficiency. The effect of liposome encapsulation on viral transduction efficiency was also studied using adenovirus encapsulated in DOTAP-folate liposomes (DfAd). While previous studies demonstrate the promising effects of DfAd in a variety of tumors [27–30], the effect of heterogenous spatial expression of CAR in IDC and ILC tumors on adenoviral therapy has yet to be investigated. The present study characterized the spatial expression of CAR in a large cohort of patient-derived IDC and ILC samples and evaluated the effects of CAR expression on adenoviral transduction. This study also characterized the expression and localization of the adherens junction proteins, E-cadherin and p120-catenin, in relation to CAR expression in IDC and ILC.

## Materials and methods

### Reagents and cell lines

Replication-deficient adenovirus expressing green fluorescent protein (Ad-GFP) was purchased from Baylor College of Medicine (Catalog: Ad5-CMV-eGFP). MCF7 cell line was from the laboratory of Dr. Tony Reid. The SUM44PE cell line was purchased from BioIVT (Catalog: HUMANSUM-0003016). Dulbecco’s modified Eagle’s medium (DMEM) with high glucose (Hyclone #SH30081.01) was supplemented with 10% fetal bovine serum (FBS, Omega Scientific #FB-01) and 1% Pen Strep Glutamine (PSG, Life Technologies #10378-016) to prepare the complete media for MCF7 cell culturing. Ham’s F-12 (Life Technologies #11765054) was supplemented with 1 g/L Bovine Serum Albumin (BSA, Sigma Aldrich #A8806), 5 mM of Ethanolamine (Sigma Aldrich #E0135), 10 mM of HEPES (Sigma Aldrich H3375), 1 µg/mL of Hydrocortisone (Sigma Aldrich #H4001), 5 µg/mL of Insulin (Sigma Aldrich #I9278), 50 nM of Sodium Selenite (Sigma Aldrich #S9133), 5 µg/mL of apo-Transferrin (Sigma Aldrich #T2252), 10 nM of Triiodo Thyronine (T3, Sigma Aldrich #T5516), and 2% FBS to prepare the complete media for the SUM44PE cell line. Rosewell Park Memorial Institute (RPMI) 1640 (Gibco #11875093) medium was supplemented with 10% FBS and 1% PSG to prepare complete RPMI medium. A 1:1 mix of complete DMEM and complete RPMI medium was prepared to culture the MDA-MB-231 cell line. Human tumor digestion buffer was prepared with DMEM/F12 + GlutaMAX (Gibco #10565018) supplemented with 10 mM HEPES, 2% BSA, 5 µg/mL insulin, 0.5 µg/mL hydrocortisone, and 50 µg/mL gentamycin. Human tumor digestion buffer was also prepared using RPMI 1640 and the human tumor dissociation kit (Miltenyi Biotec #130-095-929).

Primary human breast cell medium was prepared by supplementing DMEM/F12 (1:1) with HEPES (HyClone #SH30023.01) with 10 mM HEPES (Sigma #H3537), 5% FBS, 1 mg/mL bovine serum albumin (BSA, Sigma #A7906), 1 µg/mL insulin (Invitrogen #51500-056), 0.5 µg/mL hydrocortisone (Sigma #H0888), 50 µg/mL gentamycin (HyClone #3V30080.01), and 2.5 µg/mL Fungizone. Primary human breast cell media was also prepared using EpiCult™-C Human Medium Kit (Stemcell™ Technologies #05630) by following the kit instructions for preparation of the complete EpiCult™-C Medium. Anti-CAR antibody (polyclonal, #PA5-110995) was purchased from Invitrogen, Anti-E-Cadherin antibody (clone 24E10, #3195P) was purchased from Cell Signaling Technology, and Anti-p120-Catenin (clone 98, #790–4517) was purchased from Roche Diagnostics. Anti-Rabbit HRP Polymer (#2RH-100) and Anti-Mouse HRP Polymer (#2MH-100) were obtained from Cell IDX. Tyramide-488 Reagent (#B40953), Tyramide-647 Reagent (#B40958), and Tyramide-594 Reagent (#B40957) were obtained from Thermo Fisher Scientific.

### Synthesis of liposome-encapsulated Ad-GFP

Liposome-encapsulation of Ad was also performed by extrusion, which was described previously [28]. In brief, 1,2-dioleoyl-3-trimethylammonium-propane (DOTAP, Avanti #890890 C), cholesterol (Sigma #C3045), 1,2-distearoyl-*sn*-glycero-3-phosphoethanolamine-N-[carboxy(polyethylene glycol)-2000] [PEG(2000)-PE carboxylic acid, Avanti #880124P], and 1,2-distearoyl-*sn*-glycero-3-phosphoethanolamine-N-[folate(polyethylene glycol)-2000] [PEG(2000)-Folate-PE, Avanti #880124P] were mixed together in chloroform at a molar ratio of 1:0.26:0.02:0.01. To make 400 uL of DOTAP-folate Ad-GFP (DfAd-GFP), 387 nmol of DOTAP, 100 nmol of Cholesterol, 7.01 nmol of PEG(2000)-PE carboxylic acid, and 3 nmol of PEG(2000)-folate-PE was added to 193.1 µL of chloroform (Sigma #C2432) in an amber vial (Fisher Scientific #03-339-23 C). The lipid mixture was vortexed for 30 min at 25 ℃. Subsequently, the mixture was vortexed in an amber vial for 30 min at room temperature. The resulting mixture was vacuumed overnight to form a dry lipid film at the bottom of the vial. The next day, the dry film was rehydrated with 400 uL of phosphate buffered saline (PBS, Fisher Scientific #10010072) while vortexing. The hydrated film was stirred at 600 rpm for 30 min at 4 ℃. Empty liposomes were formed by extruding the lipid mixture with the Avanti Mini Extruder (Avanti #6100009-1EA) through a 200 nm membrane (Cytiva/Whatman #10417004), 8 times at room temperature. To the empty liposomes, Ad-GFP was added, and the mixture was incubated at room temperature for 30 min to allow for encapsulation of the Ad-GFP, resulting in extruded DOTAP-folate Ad-GFP liposomes (DfAd-GFP). The resulting extruded DfAd-GFP has an Ad to DOTAP lipid ratio [Viral Particles (VP): nmol] of 5.17 × 10^7^. For clarification, a large numerical value for this ratio does not signify an excess of viral particles relative to lipid nanoparticles; in fact, only ∼ 10% of the liposomes encapsulate the virus [28].

### Human breast cell isolation and culturing

Human breast tumor cell isolation and culturing was described previously [30]. Biospecimens were collected by the Moores Cancer Center Biorepository and Tissue technology shared resource from consented patients under a University of California, San Diego Human Research Protections Program Institutional Research Board approved protocol (HRPP# 181755). Tumor fragments were acquired from two different areas of the same tumor when possible. During transportation, the acquired tumor tissues were placed in a 50 mL conical tube with sterile PBS such that the tissue sample was entirely submerged in PBS. 2 mg/mL of type 3 collagenase (Worthing #LS004182) and hyaluronidase (Sigma #H3884) 100 U/mL in human tumor digestion buffer were prepared. 10 mL per gram of tissue 10 mL of digestion buffer containing enzymes was added into a well in a 6-well plate. Tissue was placed into the well and minced until finely chopped. If needed, a syringe plunger was used to smash the tissue. The resultant tissue mixture was incubated at 37 ℃ and 5% CO_2_ with pipette mixing performed every 30 min until 5 h of digestion time. Tissue was also digested using the Miltenyi Biotec gentleMACS™ Octo Dissociator with Heaters following the protocol provided in the Human Tumor Dissociation Kit (#130-095-929). The “37C_h_TDK_3” program was selected on the Octo Dissociator instrument when starting the digestion.

After digestion, the tissue mixture was strained using a 100 μm strainer and the filtrate was centrifuged at 530 g at room temperature for 5 min to collect cells, and the supernatant was removed. If red cells were observed in the cell pellet, then 5–10 mL of ACK buffer (Quality Biological #118-156-101) was added and incubated for 3 min. Cells were centrifuged at 530 g at room temperature for 5 min, and the supernatant was removed. This step was repeated until the red blood cells in the pellet were not visible. The resultant cells were resuspended in 10 mL of PBS and centrifuged at 530 g at room temperature for 5 min. The resultant cells were resuspended in 1 mL of primary human breast cell medium, and an aliquot of 10 µL was used for cell counting. Cells were plated at 30,000 cells/well in a 96-well plate and incubated at 37 ℃ and 5% CO_2_. Viral infection was performed after cells attached to the well (24–48 h after plating).

### In vitro transduction

Cells were plated at 3 × 10^4^ cells/well in 96-well plates and incubated at 37 ℃ and 5% CO_2_ in complete media. Viral infection was performed after cells attached to the well (24–48 h after plating). DfAd-GFP or Ad-GFP were added to the cells (day 1) and incubated at 37 ℃ and 5% CO_2_. GFP fluorescence intensities were measured using a Tecan F PLEX Infinite 200 Pro microplate reader (Tecan Group Ltd., Männedorf, Switzerland) on day 4 for human primary breast cancer cells, MCF7, SUM44PE, and MDA-MB-231 cells.

### Fluorescence microscopy

Cells transduced with DfAd-GFP or Ad-GFP were analyzed under a Keyance BZ-X710 microscope (KEYENCE CORPORATION OF AMERICA, IL, USA) with a GFP filter and 470/40 nm excitation wavelength, 525/50 nm emission wavelength and dichroic mirror wavelength of 495 nm. Comparative micrographs were captured using 2x and 20x objective lenses.

### CAR expression analysis by immunohistochemistry

All formalin-fixed paraffin-embedded (FFPE) human breast cancer specimens were processed and stained by the Biorepository and Tissue Technology Shared Resources (BTTSR) at UCSD. Tissue samples were baked at 60 ℃ for 1 h. Tissue samples were cleaned and rehydrated through successive liquid dips: three times through xylene, two times through 100% ethanol, two times through 95% ethanol, two times in 70% ethanol, and one time in deionized water. Tissue samples underwent antigen retrieval in tris-based antigen unmasking solution (pH9, Vector Laboratories #H-3301) at 95 ℃ for 30 min. Immunohistochemical staining was performed on an Intellipath automated IHC stainer (Biocare Medical, LLC., CA, USA). Tissue samples were treated with Bloxall (Vector Laboratories, #SP-6000) peroxidase block for 10 min. The tissues were washed two times with tris-buffered saline (TBST, Santa Cruz Biotechnology #sc-36231-1). Tissue samples were blocked with 3% donkey serum in TBST for 10 min. The tissues were treated with 1:50 diluted rabbit anti-CAR primary antibody for 1 h. Afterwards, the tissue samples were washed two times with TBST. Tissues were treated with anti-rabbit HRP polymer for 30 min. Afterwards, the tissue samples were washed twice with TBST. Tissue samples were treated with brown DAB chromagen (VWR #95041-478) for 5 min. Subsequently, Tissue samples were washed with deionized water twice. Afterwards, the tissues were counterstained with Mayer’s Hematoxylin (Sigma #51275-500 ml) for 5 min. Tissue samples were then washed twice with TBST and once with deionized water. Tissues were then dehydrated, cleared, and mounted with a xylene based mounting mixture.

### Spatial analysis of CAR, p-120 catenin, and E-cadherin by multiplexing immunofluorescence

Tissue samples were baked at 60 ℃ for 1 h. Tissue samples were cleaned and rehydrated through successive alcohol dips: three times through xylene, two times through 100% ethanol, two times through 95% ethanol, two times in 70% ethanol, and one time in deionized water. Tissue samples underwent antigen retrieval in citrate-based antigen unmasking solution (pH6, Vector Laboratories #H-3300) at 95 ℃ for 30 min. Immunohistochemical staining was performed on an Intellipath automated IHC stainer. Tissue samples were treated with Bloxall peroxidase block for 10 min. The tissues were washed two times with tris-buffered saline (TBST, Santa Cruz Biotechnology #sc-36231-1). Tissue samples were blocked with Blotto (Thermo Fisher Scientific, #PI37530) protein block for 10 min. Tissues were treated with 1:50 diluted rabbit anti-CAR primary antibody for 1 h. Tissue samples were washed twice with TBST. The tissues were treated with anti-rabbit HRP polymer for 30 min. Tissue samples were washed twice with TBST. Tissue samples were treated with Tyramide-488 Reagent for 10 min. The tissues were washed twice with deionized water. Tissues underwent another cycle of antigen retrieval in citrate-based antigen unmasking solution at 95 ℃ for 30 min.

Tissues were treated with Ready-to-use Mouse Anti-p120 Catenin Primary Antibody for 1 h. Tissue samples were washed twice with TBST. The tissues were treated with anti-rabbit HRP polymer for 30 min. Tissue samples were washed twice with TBST. Tissue samples were treated with Tyramide-647 Reagent for 10 min. Tissues underwent one last cycle of antigen retrieval in citrate-based antigen unmasking solution at 95 ℃ for 30 min. Tissues were treated with 1:2000 diluted anti-E-cadherin primary antibody for 1 h. Tissue samples were washed twice with TBST. The tissues were treated with anti-rabbit HRP polymer for 30 min. Tissue samples were washed twice with TBST. Tissue samples were treated with Tyramide-594 reagent for 10 min. The tissues were washed twice with deionized water. The tissue samples were counterstained with 1 µg/mL of DAPI for 5 min. Tissues were mounted with Vectashield Vibrance (Vector Laboratories #H-1700-10) cover slips.

## Results

**Fig. 1:**
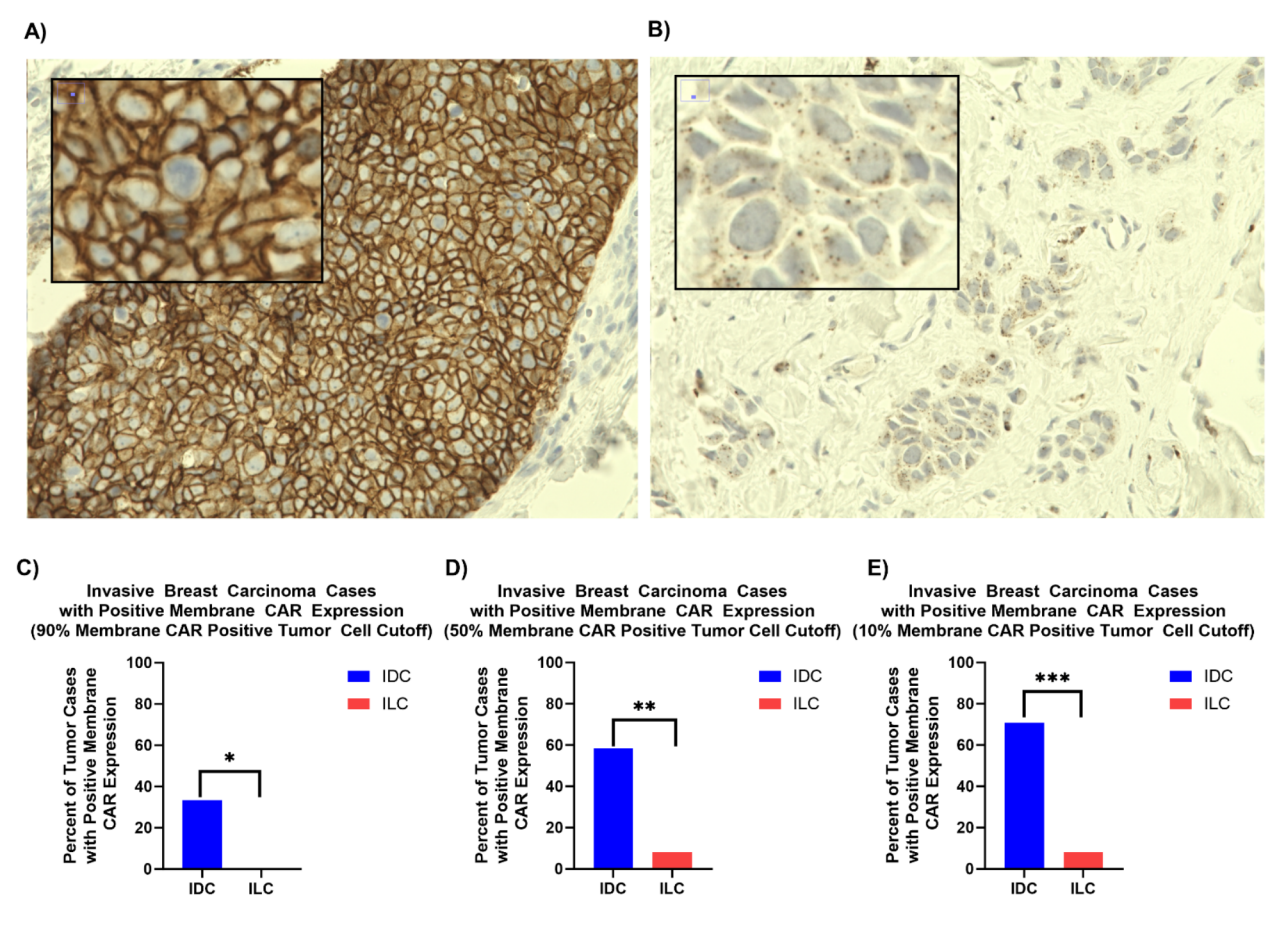
CAR expression in invasive breast cancer. (**A**) This is an example IDC case with 3 + CAR staining intensity in the membrane of the tumor cells. (**B**) This is an example ILC case where there is no membrane CAR in the tumor cells. Instead, it presents dot-like CAR aggregates in the cytosol of the tumor cells. The staining is all located inside the cells and not the intracellular space. (**C**) At a 90% positive tumor cell cutoff, there were significantly greater IDC cases with positive membrane CAR expression compared to ILC cases. *p < 0.05. (**D**) At a 50% positive tumor cell cutoff, there were significantly greater IDC cases with positive membrane CAR expression compared to ILC cases. **p < 0.005. (**E**) At a 10% positive tumor cell cutoff, there were significantly greater IDC cases with positive membrane CAR expression compared to ILC cases. ***p < 0.0005

### CAR expression analysis of invasive breast carcinoma

A total of 36 FFPE preserved patient breast cancer samples were obtained from BTTSR at UCSD. After hematoxylin and eosin (H&E) and CAR staining of the tissues, a pathologist diagnosed 24 cases as IDC and 12 cases as ILC. The pathologist also evaluated CAR expression, staining intensity, and the percentage of CAR-expressing tumor cells. Two distinct localization patterns were observed for CAR: membranous and punctate cytoplasmic. (Figure 1A and B) Out of these two spatial expression patterns, IDC cases showed CAR expression mainly in the membrane of tumor cells while ILC cases showed punctate CAR staining within the cytoplasm. The IDC cases showed positive membrane staining in 50.7% of tumor cells while ILC cases showed positive membrane staining in only 7.2% of tumor cells. (Figure S1) The percentage of tumor cells displaying CAR membrane expression in each case was further investigated and with a cutoff of 90% positive membrane-staining tumor cells, 33.3% of the IDC cases were positive for membranous CAR expression while none of the ILC cases were positive. (Fig. 1C) Even when the cutoff was set to 10%, only 8.3% of ILC cases were positive for membrane CAR expression while 70.8% of IDC cases were positive. (Fig. 1E) Therefore, the present study documents that IDC generally expresses CAR in the membrane of tumor cells while in ILC, CAR is expressed in the cytoplasm as dot-like aggregates.

**Fig. 2:**
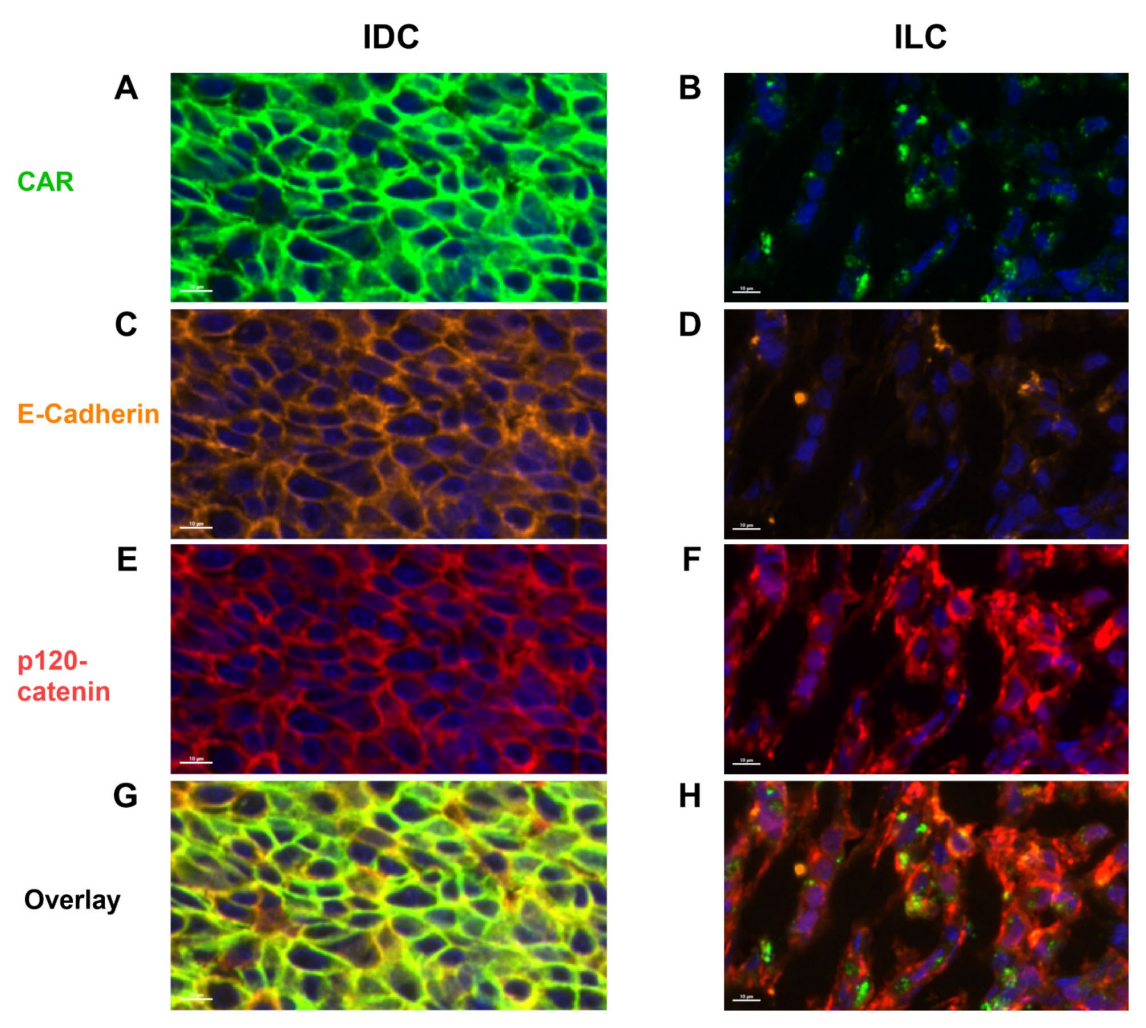
Co-expression of CAR, p120-Catenin, and E-Cadherin in invasive lobular carcinoma using multiplex immunofluorescence histology. (**A**)(**C**)(**E**)(**G**) CAR in green, E-Cadherin in orange, p120-Catenin in red, and overlap fluorescent micrographs of a human IDC tumor sample, respectively. Note that all proteins are in the membrane of IDC tumor cells. Magnified 89.2x (**B**)(**D**)(**F**)(**H**) CAR in green, E-Cadherin in orange, p120-Catenin in red, and overlap fluorescent micrographs of a human ILC tumor sample, respectively. Note that both CAR and p120-catenin are in the cytoplasm of ILC tumor cells. Magnified 82.9x

### Co-expression of CAR, E-cadherin, and p120-catenin

To investigate the spatial localization of CAR in relation to other junction proteins, three IDC and three ILC tissue blocks from the CAR expression analysis in Fig. 1 were sectioned and assayed with a multiplex immunofluorescence panel of anti-CAR, anti-E-cadherin, and anti-p120-catenin antibodies. All three IDC cases demonstrated co-expression of CAR, E-cadherin, and p120-catenin in the membrane of tumor cells. An example of the membranous co-expression of all three biomarkers in IDC is shown in Fig. 2A, C and E, and G, and lower magnification images are shown in the supplement (Figures S2A, C, E, G). In all three ILC cases, CAR and p120-catenin are both located in the cytoplasm; there is weak cytoplasmic fluorescence in the E-cadherin channel, but it may be attributed to bleed-through by the p120-catenin fluorophore (Fig. 2B, D and F, and H, and supplemental Figures S2B, D, F, H).

**Fig. 3:**
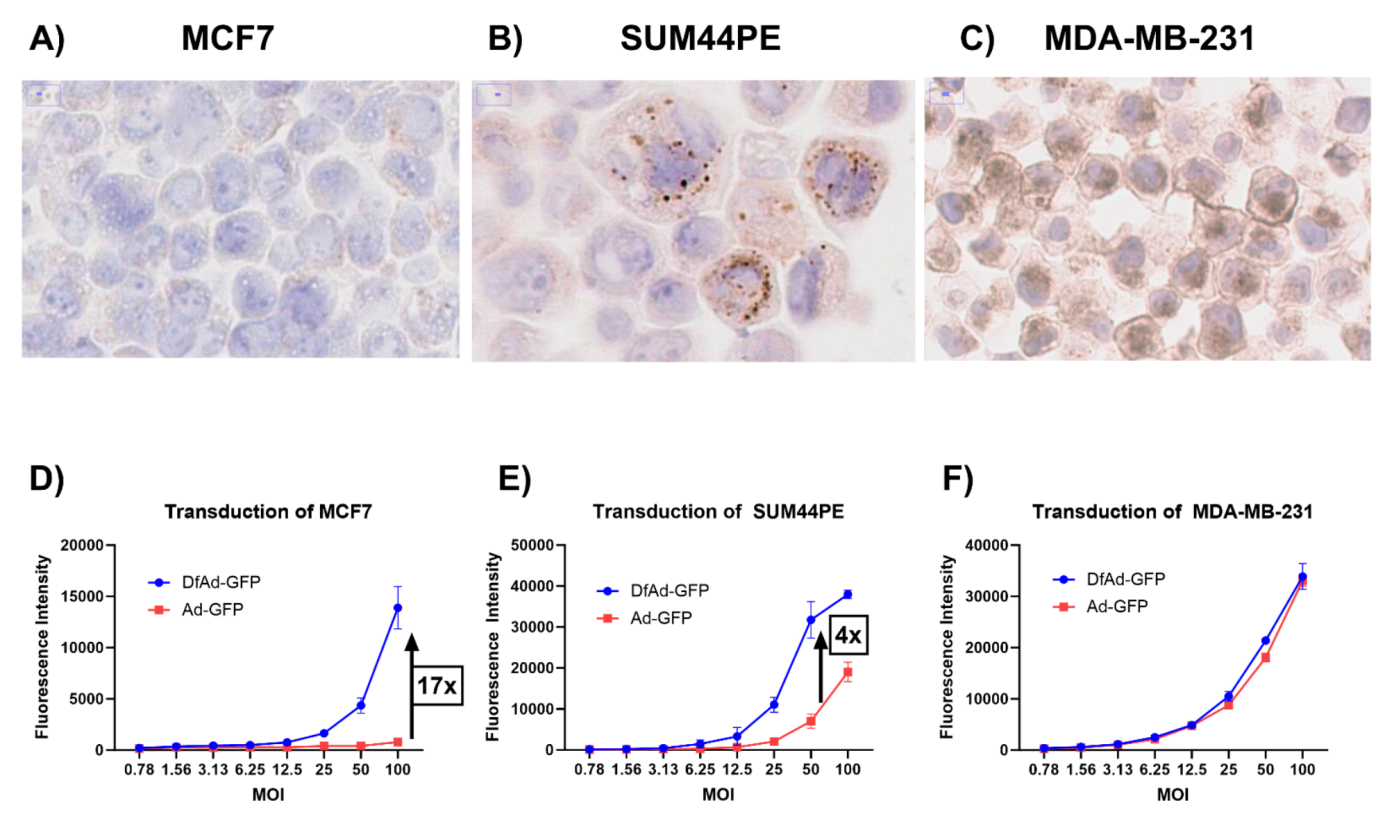
In vitro transduction of IDC and ILC cell lines. (**A**) CAR IHC of MCF7 shows negative CAR expression in membrane and cytoplasm. (**B**) CAR IHC of SUM44PE shows negative CAR expression in membrane and punctate cytoplasmic expression. (**C**) CAR IHC of MDA-MB-231 show positive CAR expression in membrane and some cytoplasmic expression. (**D**) Transduction efficiency of MCF7, a CAR-negative IDC cell line. (**E**) Transduction efficiency of SUM44PE, a membrane CAR-negative ILC cell line. (**F**) Transduction efficiency of MDA-MB-231, a membrane CAR-positive IDC cell line. Error bars are standard error of the mean (SEM)

### Transduction of established IDC and ILC Cell lines with DfAd and ad

To learn how CAR expression differences affect the transduction efficiency of each of the cell lines in this study, CAR IHC staining was performed on cell pellets of each of the cell lines. The cell lines used were MCF7, a CAR-negative IDC cell line, MDA-MB-231, a high-CAR IDC cell line and SUM44PE is an ILC cell line. CAR IHC staining reveals no CAR expression in either the membrane or cytoplasm in MCF7 (Fig. 3A). CAR expression is dot-like in the cytoplasm of SUM44PE similar to that observed in human ILC tissue specimens (Fig. 3B). CAR IHC staining of MDA-MB-231 demonstrated high CAR expression in the cell membrane similar to what was observed in human IDC tissue specimens (Fig. 3C). To model how DfAd and Ad transduce IDC and ILC tumor cells, MCF7, SUM44PE, and MDA-MB-231 cells were transduced by DfAd-GFP and Ad-GFP. MCF7 cells treated with DfAd-GFP at MOI 100 demonstrated 17-times higher transduction efficiency compared to cells treated by Ad-GFP at the same MOI (Fig. 3D). SUM44PE cells treated with DfAd-GFP at MOI 50 demonstrated 4-fold higher transduction efficiency compared to cells treated by Ad-GFP at the same MOI (Fig. 3E). MDA-MB-231 cells treated with DfAd-GFP demonstrated similar transduction efficiencies to cells treated by Ad-GFP at all MOIs (Fig. 3F).

**Fig. 4:**
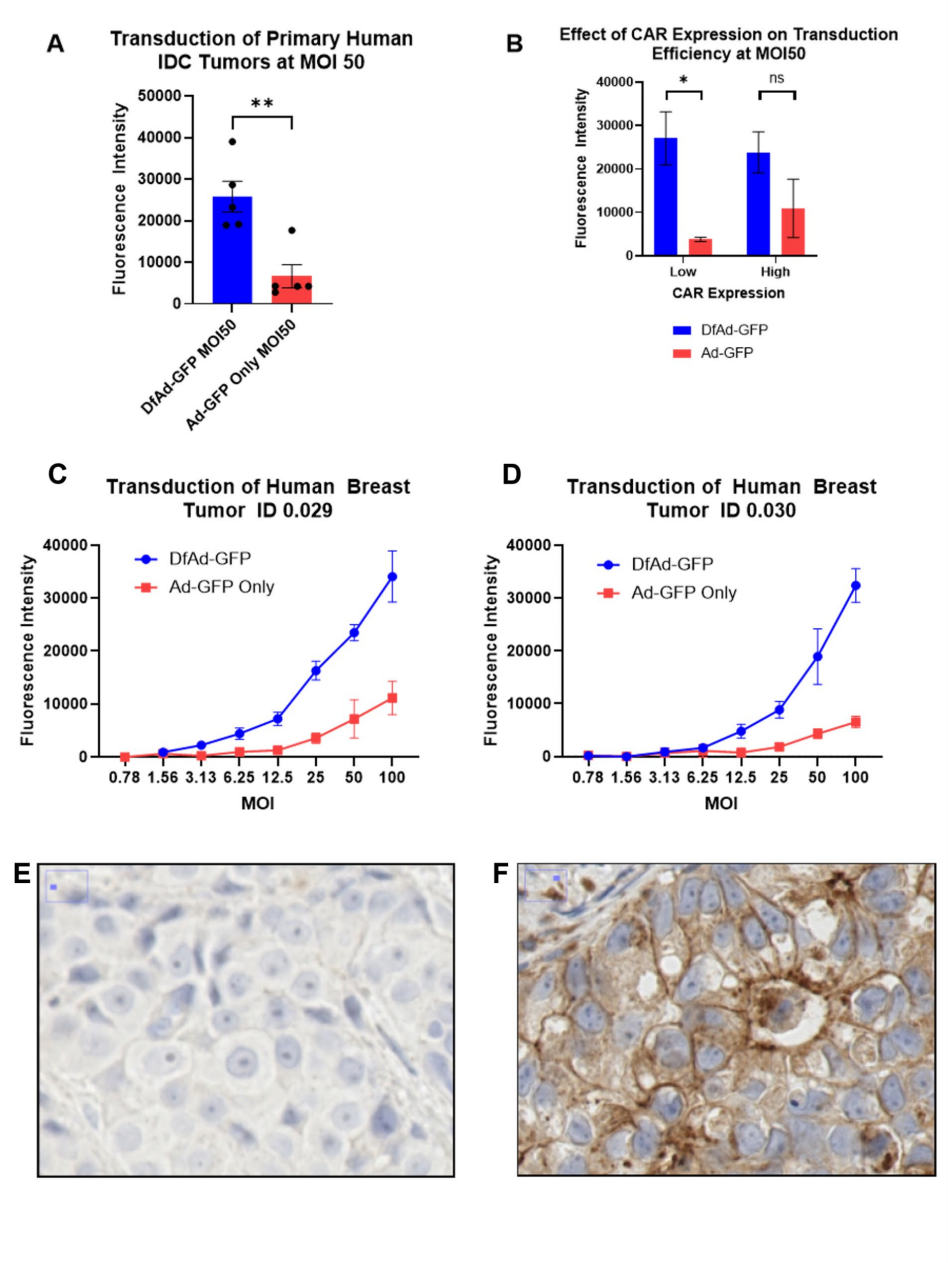
In vitro transduction of patient derived IDC tumor samples. (**A**) The average transduction efficiency of primary human IDC tumors when transduced with DfAd-GFP versus Ad-GFP at MOI 50. *n* = 5, **p < 0.005 (**B**) Effect of CAR Expression on transduction efficiency of DfAd-GFP to Ad-GFP of primary human IDC tumors after batching cases with staining intensity 0–2 into “Low” CAR expression and 3 into “High” CAR Expression (*n* = 3 in Low and *n* = 2 in High), *p < 0.05, ns = not significant. (**C**) The transduction efficiency of primary human tumor sample ID 0.029 when transduced with DfAd-GFP versus Ad-GFP at multiple MOI. *n* = 3. (**D**) The transduction efficiency of primary human tumor sample ID 0.030 when transduced with DfAd-GFP versus Ad-GFP at multiple MOI. *n* = 3. Of the five reported tumor samples, 0.029 and 0.030 were the only two samples that yielded sufficient cells to measure transduction efficiency at multiple MOIs. (**E**) CAR IHC staining of an IDC tumor that shows CAR staining intensity < 1+. (**F**) CAR IHC staining of an IDC tumor that shows very high CAR staining intensity 3+. Error bars are SEM

### Transduction of human primary IDC tumor cells

To model how DfAd and Ad transduce primary human breast tumors, human breast tumor samples were homogenized into single cells and transduced with DfAd-GFP and Ad-GFP. Data from five unique IDC tumor cases were collected by the end of this study. The average transduction efficiency of all five breast tumors treated with DfAd-GFP at MOI 50 was about 4-fold higher than when they were treated with Ad-GFP at MOI 50 (Fig. 4A). To learn how CAR expression differences affect the transduction efficiency of each of the breast tumor samples, a pathologist quantified the membranous and cytoplasmic CAR expression for each tumor sample by evaluating CAR IHC-stained tissues for each of the tumor cases. Human IDC tumor samples with low CAR expression demonstrated 7-fold higher transduction efficiency when treated with DfAd-GFP compared to when treated with Ad-GFP (Fig. 4B). However, human IDC tumor cases with high CAR expression only demonstrated 2-fold higher transduction efficiency when treated with DfAd-GFP compared to when the cells were treated with Ad-GFP, but this difference was not statistically significant (Fig. 4B). DfAd-GFP demonstrated higher transduction efficiency than Ad-GFP in two human breast tumor samples at MOI 12.5 or greater (Fig. 4C-D). IHC of one of the human primary IDC tumor samples with low CAR expression is shown in Fig. 4E. IHC of a tumor sample with high membranous CAR expression is shown in Fig. 4F. Unbinned data showing the transduction efficiencies and CAR staining intensities of each tumor case is shown in the supplement (Figure S3).

**Fig. 5:**
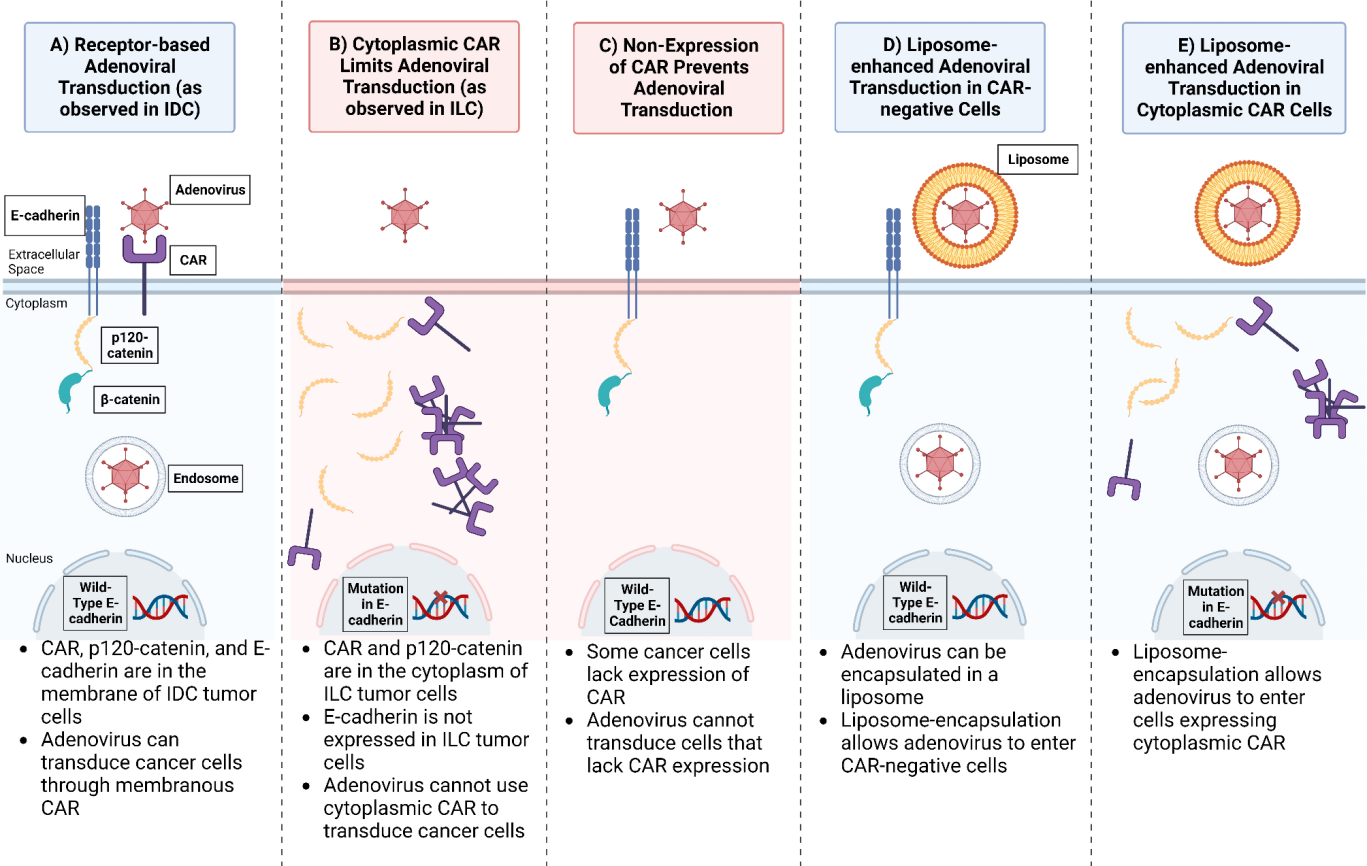
Schematic of adenoviral transduction and CAR-catenin-cadherin expression in invasive breast carcinoma. (**A**) In IDC, wild-type E-cadherin is expressed in the membrane of breast cancer cells, and becomes complexed with p120-catenin and β-catenin. CAR is also expressed on the membrane of breast cancer cells. Breast cancer cells expressing membrane-bound CAR can be transduced by adenovirus through receptor-based transduction. (**B**) In ILC, the E-cadherin gene is mutated resulting in loss of expression of E-cadherin, resulting in p120-catenin translocation to the cytoplasm. CAR is also translocated to the cytoplasm. Breast cancer cells that only express cytosolic CAR cannot be transduced by the adenovirus. (**C**) Breast cancer cells that do not express any CAR cannot be transduced by adenovirus. This is the scenario when transducing ILC and IDC tumor cells that do not show CAR expression. (**D**) Without CAR expression, liposome encapsulation is used to allow adenovirus entry into the cell without needing CAR. (**E**) To enter cells expressing only cytoplasmic CAR, liposome encapsulation is used to allow adenovirus entry into the cell without needing membranous CAR

## Discussion

CAR is a transmembrane protein and makes up tight junctions between epithelial cells as part of the junction adhesion molecules. While CAR expression in breast cancer has been previously studied, this study is the first report to demonstrate the spatial heterogeneity of CAR in IDC and ILC, as well as dot-like, cytoplasmic spatial expression pattern of CAR in the ILC samples. Martin et al. measured CAR mRNA expression in various breast cancers by reverse transcription quantitative polymerase chain reaction (RT-qPCR) and found that ductal carcinoma showed elevated transcriptional CAR expression compared to lobular carcinoma [38]. Although they also show some micrographs of CAR IHC staining of breast tumor tissues, the lack of contrast between the CAR stain and the nuclear stain, and the grey scale coloration make the IHC results difficult to interpret [38]. Reeh et al. measured CAR expression by CAR IHC staining of microarrays in a broad range of tumors [41]. Despite having a larger sample size (*n* = 60 IDC and *n* = 64 ILC), they demonstrated much lower percentages of CAR positive IDC cases (8.3%) compared to the present study. (41) This discrepancy may be caused by heterogeneous expression of CAR in breast tumors as tissue microarrays (TMAs) only sample a small portion of each tumor block for interpretation. The advantage of whole tumor section evaluation is the potential to better interpret and evaluate heterogeneous tumors. It is noted that Reeh et al. used different antibodies and methodology for their IHC, potentially resulting in different tissue staining results. They also analyzed their pathology results using “immunoreactive score” instead of staining intensity, likely resulting in a different distribution of positive and negative cases. Furthermore, the Reeh study did not perform an in-depth analysis of CAR localization patterns in IDC compared to ILC as we have done in the present study.

One of the pathophysiological characteristics of ILC is the mutation in the *CDH1* gene, resulting in the loss of E-cadherin expression in the epithelial cells [42, 43]. In response to this, p120-catenin translocates from the membrane to the cytoplasm, which initiates ILC tumor progression and increases tumor cell invasion [44–47]. Just as the loss of E-cadherin results in the cytoplasmic localization of p120-catenin, we observed that the loss of E-cadherin in the ILC correlates with the cytoplasmic localization of CAR (illustrated in Fig. 5). Previous studies investigated how CAR regulates E-cadherin stability in model cell lines in vitro [48, 49], however, our study is the first to show that the loss of E-cadherin, as observed in ILC, affects CAR localization. Figure S4 summarizes how membrane expression levels of CAR, E-cadherin, and p120-catenin differ between IDC and ILC.

Using cell lines MCF7 and MDA-MB-231 to model IDC with no CAR expression and high membrane CAR expression, respectively, we show that liposome encapsulation (DfAd-GFP) increases adenoviral transduction efficiency up to 17-fold in CAR negative cells. In contrast, the high-CAR cell line, MDA-MB-231 showed no change in transduction efficiency by liposome encapsulation as Ad-GFP could already enter the cells efficiently through the CAR receptor. Previous literature had used other CAR-negative and CAR-positive IDC cell lines to evaluate DfAd and Ad transduction efficiency. (28–30) However, no previous studies had modeled DfAd and Ad transduction in ILC cell lines. Our experiments using SUM44PE, an ILC cell line, showed 4-fold enhanced transduction efficiency using DfAd-GFP compared to Ad-GFP, indicating that liposome enhanced transduction is more efficient in ILC tumor cells than receptor-based Ad transduction through CAR. In primary human IDC tumor samples, DfAd-GFP demonstrated 4-fold higher transduction efficiency compared to the Ad-GFP, indicating that liposome encapsulation can improve Ad transduction in human IDC tumor cells. This differential may improve adenoviral mediated therapy for human breast tumors. Our study was limited by the absence of primary ILC tumor specimens; we plan future studies using human ILC patient-derived xenograft models in mice to study the treatment of human ILC using liposome-encapsulated adenoviral therapy.

## Conclusion

Oncolytic adenoviral therapy is a potentially promising therapeutic option for breast cancer patients. The present study demonstrated that the two major subtypes of invasive breast carcinoma, IDC and ILC, differ significantly in terms of spatial expression of CAR and adenoviral transduction efficiency, which may impact the efficacy of adenoviral-based therapeutics. While IDC tumors typically express CAR in the membrane of tumor cells, ILC tumors express CAR mainly in the cytoplasm. We show that liposome encapsulation improves adenoviral transduction in ILC cell lines expressing cytoplasmic CAR. We also observed that in human primary breast cancer cells, liposome encapsulation (DfAd) demonstrated higher transduction efficiency compared to unencapsulated adenovirus (Ad). The present study also showed a correlation between CAR, E-cadherin, and p120-catenin in IDC and ILC in human breast cancer tissue, which suggests an interdependency of tight and adherens junction proteins. Overall, our study suggests that adenoviral therapy would most likely benefit patients with CAR-positive IDC tumors, while liposome-enhanced adenoviral delivery would be needed patients with ILC or CAR-negative IDC. This novel immunotherapy extends therapeutic opportunities for ILC patients which primarily consists of hormonal therapy at present and has the potential for rapid translation to clinical use.

## Electronic supplementary material

Below is the link to the electronic supplementary material.

Supplementary Material 1

## Data Availability

All datasets used and analyzed during the current study are available from the corresponding author on reasonable request.

## Abbreviations

Ad: Adenovirus
BSA: Bovine serum albumin
CAR: Coxsackievirus & adenovirus receptor
DfAd: DOTAP-folate liposome encapsulated adenovirus
DMEM: Dulbecco’s Modified Eagle Medium
DOTAP: 1,2-dioleoyl-3-trimethylammonium-propane
ER: Estrogen receptor
FBS: Fetal bovine serum
FFPE: Formalin-fixed paraffin-embedded
GFP: Green fluorescent protein
H&E: Hematoxylin and eosin
IDC: Invasive ductal carcinoma
IHC: Immunohistochemistry
ILC: Invasive lobular carcinoma
MOI: Multiplicity of infection
PBS: Phosphate buffered saline
PEG(2000)-Folate-PE: 1,2-distearoyl-sn-glycero-3-phosphoethanolamine-N-[folate(polyethylene glycol)-2000
PEG(2000)-PE carboxylic acid: 1,2-distearoyl-sn-glycero-3-phosphoethanolamine-N-[carboxy(polyethylene glycol)-2000
PSG: Penecillin-streptomycin-glutamine
RPMI: Roswell Park Memorial Institute
RT-qPCR: Quantitative reverse transcription polymerase chain reaction
T3: Triiodo thyronine
TBST: Tris-buffered saline Tween 20
TMA: Tumor microarray
VP: Viral particle
