# Supplementary material for: CAR expression in invasive breast carcinoma and its effect on adenovirus transduction efficiency
Source: Breast Cancer Res. 2024 Sep 10;26:131. doi: 10.1186/s13058-024-01880-z (PMC11389499; doi:10.1186/s13058-024-01880-z)
Supplement: Supplementary file 1 — Supplementary Material 1 [file 13058_2024_1880_MOESM1_ESM.docx]

**Supplementary Figures**


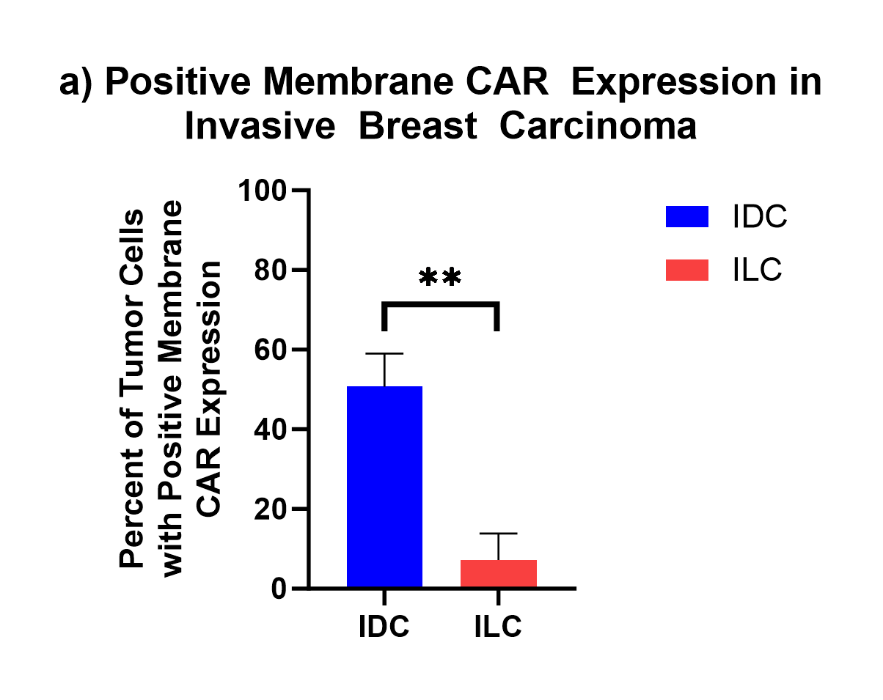


Supplement Figure 1. IDC tumors, on average, showed significantly higher percentages of tumor cells that stained for CAR at 1-3+ intensity in the membrane compared to ILC tumors. **p<0.005. Error bars are SEM.


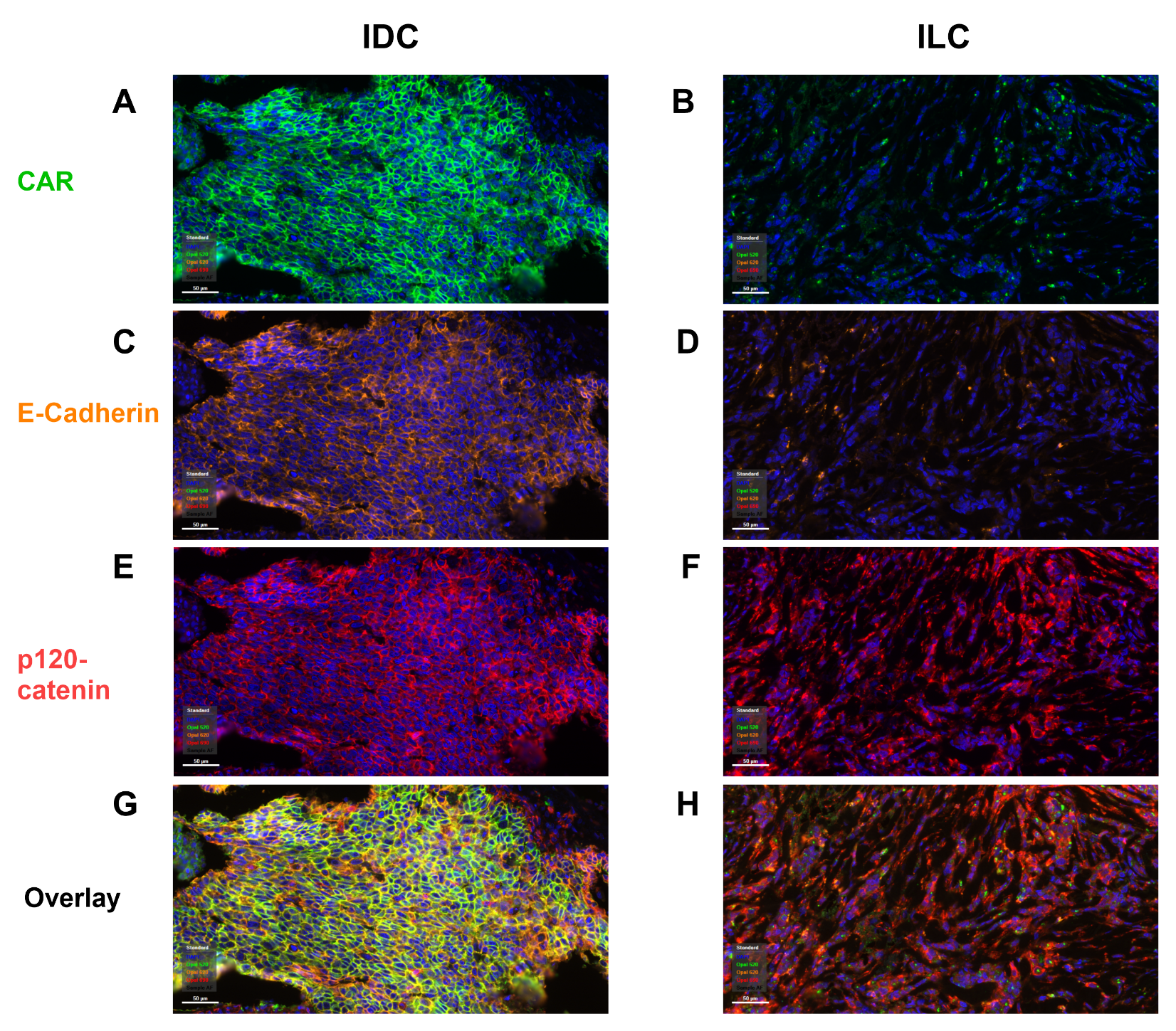


Supplement Figure 2. **(Zoomed Out) Co-Expression of CAR, p120-Catenin, and E-Cadherin in Invasive Lobular Carcinoma using Multiplex Immunofluorescence Histology.** (A)(C)(E)(G) CAR, p120-Catenin, E-Cadherin, and overlap fluorescent micrographs of a human IDC tumor sample, respectively. (B)(D)(F)(H) CAR, p120-Catenin, E-Cadherin, and overlap fluorescent micrographs of a human ILC tumor sample, respectively.


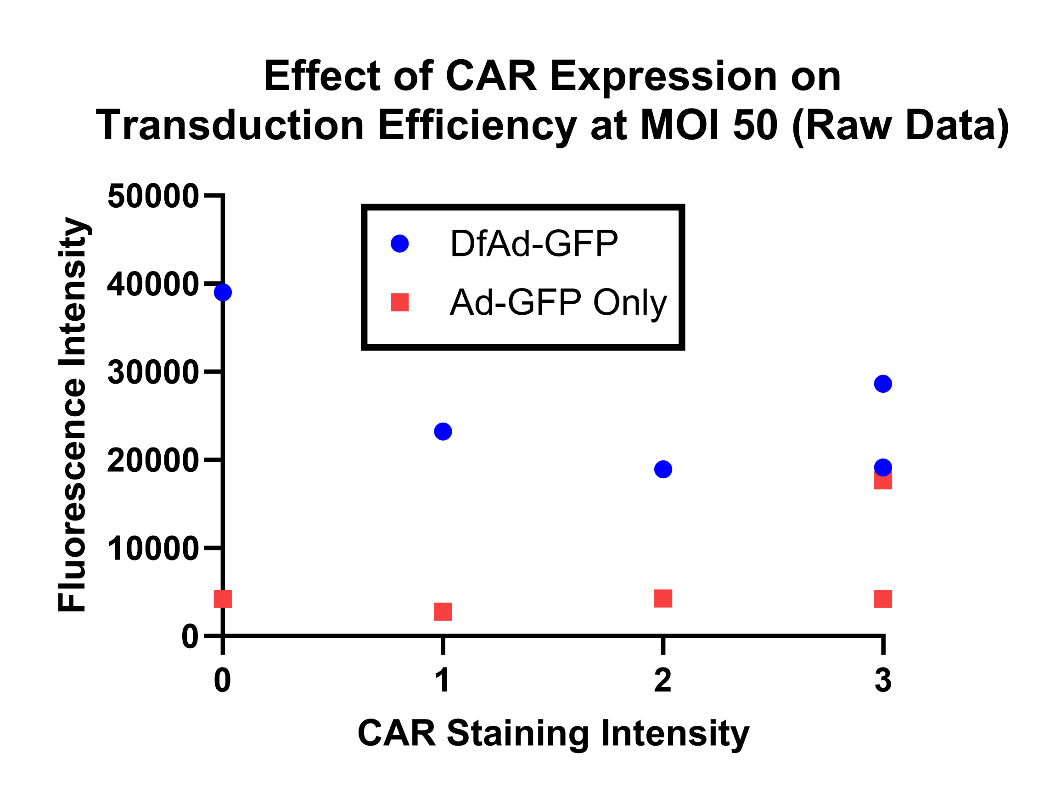


Supplement Figure 3. Effect of CAR Expression on the transduction efficiency of primary human IDC tumors.


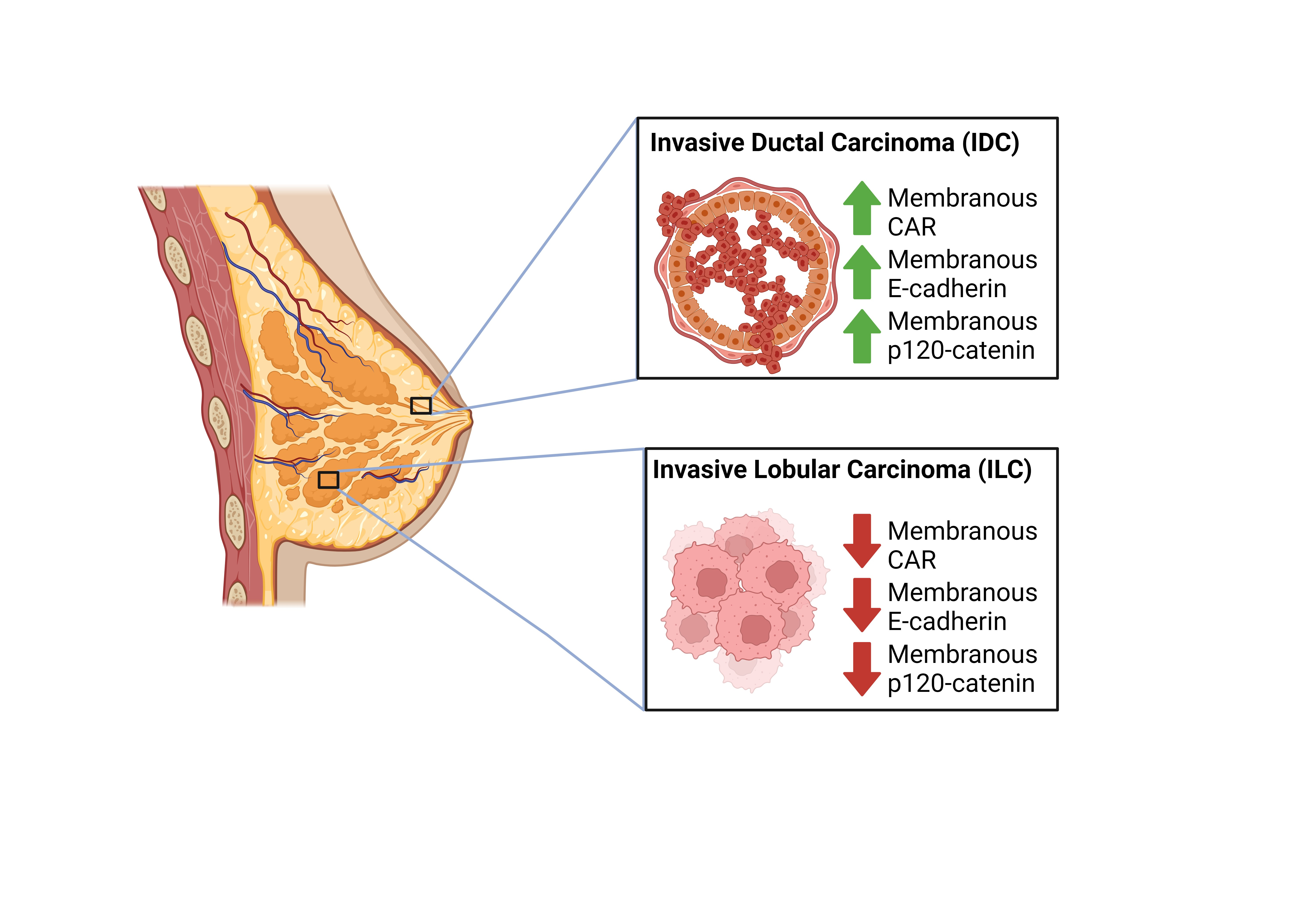


Supplement Figure 4. **Summary of pathological characteristics of invasive breast carcinoma.** IDC typically demonstrates positive E-cadherin and p120-catenin in the membranes of tumor cells. ILC is pathologically characterized by loss of expression of E-cadherin and loss of expression or translocation of p120-catenin to the cytosol. CAR expression analysis using immunohistochemistry reveals the IDC also typically expresses membranous CAR while in ILC, CAR expression is either completely lost or it is translocated to the cytosol.
